# Determination of Osteoblast Cell Viability and Histological Changes of Samples Obtained from Different Implant Drills during Osteotomy

**DOI:** 10.30476/dentjods.2022.91439.1579

**Published:** 2023-06-01

**Authors:** Janet Moradi Haghgoo, Masoume Khoshhal, Shahram Sharifi, Iraj Khodadadi, Hamidreza Ghadidmi Pour, Mohammad Ali Seif Rabie, Nazli Rabienejad

**Affiliations:** 1 Dept. of Periodontics, School of Dentistry, Hamadan University of Medical Sciences, Hamadan, Iran; 2 Periodontist, Hamadan, Iran; 3 Dept. of Biochemistry, School of Medicine, Hamadan University of Medical Sciences, Hamadan, Iran; 4 Dept. of Pathology, Hamadan University of Medical Sciences, Hamadan, Iran; 5 Dept. of Sociable Medicine, School of Medicine, Hamadan University of Medical Sciences, Hamadan, Iran

**Keywords:** Bone grafting, Particle size, Dental implant, Cell viability, Osteotomy

## Abstract

**Statement of the Problem:**

The bone particles collected during osteotomy could be used as autogenous bone graft materials for dental implant surgery. Different factors such as drill design may influence its clinical viability.

**Purpose:**

This study examined the effect of drill design on the osteoblast viability and histopathology parameters of bone collected during the preparation of dental implant site.

**Materials and Method:**

In this experimental study, 90 samples were obtained from three different bone drilling systems including Bego, Implantium, and Dio during fixture installation in patients requiring treatment at the Department of Periodontology, Dentistry University Hamedan. The MTT (3-4,5-Dimethylthiazol2,5-diphenyltetrazolium bromide) was used to determine percentage of cell viability. Samples were fixed in 10% formaldehyde for histological evaluation. Then, they were kept in 10% EDTA solution for 4 weeks for decalcification. The provided slides were evaluated regarding bone structure and osteocytes counts for assessment of viability. Tukey test and SPPS 21 software were used for statistical analysis.

**Results:**

The result showed the viability of osteoblast obtained by Dio (0.45±0.04) was significantly better than Bego (0.37±0.05) and Implantium (0.37±0.04) systems. In histopathological evaluation, the grafting material obtained by Dio presented the best osteoblast morphology.

**Conclusion:**

It might be concluded that drill geometry has significantly influenced the viability of bone particles collected during the preparation of implant sites .Moreover, characteristic geometry alone cannot represent the performance of a particular drill, and several geometric features should be concerned. The results of this study showed that the geometry of the Dio drill was the best considering the viability and histopathological evaluations.

## Introduction

There is an increasing demand for dental implant placement considering patients satisfaction in recent years. This increase might be related to particular reasons rather than replacing lost teeth; including esthetic aspects, speech ability, chewing, self-confidence, and better life quality. On other hand, dentists are interested in dental implants since there is no need to prepare tooth, and increased retention, and stability especially in fully edentulous cases are among the advantages of this treatment modality [ 1 - 2 ]. The success of implant treatment is related to adequate available bone at the implant site and this issue would influence the long-term prognosis. In addition, there are some techniques to increase the width and height of alveolar ridge including distraction osteogenesis, grafting, and bone splitting, and guided bone regeneration (GBR). Currently, autogenous bone graft is known as the gold standard for GBR [ 3 - 4 ]. 

The type of surgical drill and drilling method are the most important items in dental implant treatment. Eriksson and Albrektson [ 5 ] reported that bone becomes more damaged during drilling especially concerning thermal damage. They reported that the threshold level of bone vitality is 47°C during drilling and less than one minute. Decreased cutting efficacy and frictional heat are the consequences of using cutting instruments frequently [ 5 ]. The heat produced during drilling is related to pressure, shape, and the size of drill as well as drilling time. The factors related to heat production are reported as drilling speed, acquired depth, geometry of drill, and sharpness of drill [ 6 ].

In recent years, the use of stainless steel drills has been successful in dentistry [ 7 ]. Ceramic drills have some advantages like resistance against high temperature, corrosion, abrasion, and lower tendency for reaction with chemical materials. Despite all these, the application of this kind of drills is limited because of their lower resistance against mechanical shocks, potential fracture tendency, and low thermal conductivity [ 7 - 8 ]. It is reported that the efficacy of a drill is related to design and mechanical properties. Employment of different drill types would result in different properties of collected bone [ 9 ]. The osteoblasts create the protein matrix of bone structure. These cells usually construct bone while the osteoclasts are responsible for constant bone remodeling [ 10 ]. 

An *in vitro* study showed that bone milling resulted in decreased osteoblast cells [ 11 ]. Therefore, basic conditions for cell viability are drill selection, proper operation, and control bacterial infection. The utilization of different drills alters the osteoprotegerin [ 12 ]. Osteoprotegerin is a protein secreted from osteoblasts. Different drills usage also causes changes in receptor activator of nuclear factor kappa-Β ligand (RANKL) protein, which plays an important role in bone dynamic. RANKL is important for osteoclastic activity and bone remodeling cycle. Therefore, the type of drill is considered as an imperative factor [ 12 ].

Concerning the limited evidence on this topic, this study was conducted to assess the osteoblasts viability when different drill designs are used during osteotomy. 

## Materials and Method

In this experimental study, 90 samples were collected using three different drilling systems (30 samples for each drill type) from patients with edentulous ridge who were referred for tooth implant surgery. 

The inclusion criteria were defined as negative history of any periodontal disease and presence of good oral hygiene. The exclusion criteria were delineated as pregnancy, smoking or alcohol consumption, uncontrolled systemic disease, unacceptable oral hygiene, history of radiotherapy or chemotherapy, taking immunosuppressive medications, and long-term steroid therapy. 

The Misch classification was used to define the bone types. Correspondingly, D2 was considered as dense cortical bone with large coarse trabecular bone, and D3 was considered as thin cortical bone and dedicated trabecular bone pattern. The density of all collected samples was the same regarding the tactile sensation [ 13 ].

**Figure 1:**
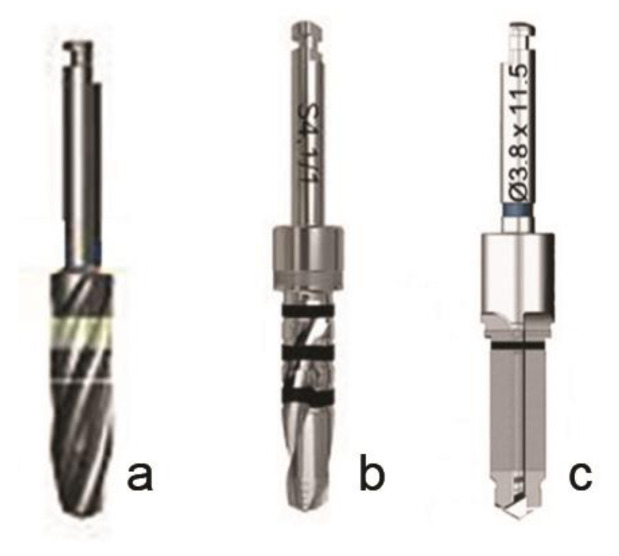
The used drills of different implant systems (**a:** Implantium, **b:** Bego, **c:** Dio)

As illustrated in Figure 1, Dio (Dio co., DAEGU, South Korean), Implantium (Dentium co., Chongju, Korea), and Bego (Bego co., Bremen, Germany) drills were employed in this study. Drilling speed was 800 rpm and equal for all groups. Patients used 0.1% chlorhexidine rinse for 2 minutes before surgery in order to reduce bacterial contamination. The collected bone samples were kept in Normal Saline solution (0.90% w/v) and then sent to laboratory on ice [ 14 ].

The MTT (3-4,5-Dimethylthiazol2,5-diphenyltetraz-olium bromide) was used to determine the percentage of cell viability. MTT is a sensitive method for evaluating osteoblasts proliferation and cell viability through oxidation of MTT by mitochondrial dehydrogenase. The Cell Titer 96 (promega, madison, WI, USA) (MTC) was used to determine the cell viability [ 15 ].

The samples were fixed in 10% formaldehyde for histological evaluation. Then, they were kept in 10% EDTA for 4 weeks for decalcification; the EDTA solution was being changed weekly. The provided slides were evaluated for bone structure and osteocytes count, which indicate the viability. 

Tissue processing was done manually after sample fixation with 10% formalin. The samples were softened and decalcified with 10% nitric acid for one hour. All samples were then rinsed to exclude the acid. 

The samples were kept at five different ethylic alcohol solutions (50%, 70%, 80%, 96%, and 100%) respectively, in order to dehydrate the excess water of fixation stage. Then, for clearing, all samples were kept in Xylenol for one to two hours and this was repeated with the new Xylenol. In impregnation stage, the samples were settled in melted paraffin at 56°C for 24 hours. The paraffin was changed to achieve better impregnation. Then, the samples were transferred at embedding stage. The utilized mold was made of aluminum. Rotary micro tom (SLEE) was used for sectioning at 5μm width serial section. Sections were floated at 48-50°C water in a dark barrel to distinguish wrinkle tissue better. Sections were located at slide impregnated with albumin adhesive. Then, slides were located on the hot plate to dry. Hematoxylin-Eosin was used for staining. Slides were evaluated with light microscope after mounting. 

Tukey test and SPPS 21 software were used for statistical analysis.

## Results

A total of 90 patients, 53 male and 37 female individuals, were enrolled in this study and divided into 3 groups. All the operations were performed by a single periodontist in posterior regions. All collected samples with different drills were examined for histopathological and viability assessment. 

### Cells viability evaluation

The viability was statistically significant in Dio group compared to other groups (*p* value 0.001) (Table 1).

**Table 1:** Comparing mean and viability among study groups

Study Groups	Mean ± SD
Dio	0.45±0.04
Bego	0.37±0.05
Implantium	0.37±0.04

As the result of Tukey test, there was a significant difference among Dio drill samples and the others in favor of viability (*p* value 0.001). However, there were not any significant differences between Bego and Implantium drill samples (*p* value 0.948 for differences between Bego and Implantium). As illustrated in Table 2, of the t-test revealed that the quality of 42% of samples was D2 and 58% was D3. The viability values were significantly more for D3 compared with D2 in all study groups. Dio drill samples have been shown to present the most viability values (Table 2). 

**Table 2:** Comparing viability among study groups for D2 and D3

Study Groups	Bone type	Number	Mean±SD	*p* Value
Dio	D2	13	0.42±0.03	0.001
	D3	17	0.48±0.04	
Implantium	D2	11	0.37±0.05	0.029
	D3	19	0.43±0.03	
Bego	D2	14	0.33±0.03	0.001
	D3	16	0.41±0.05	

Based on the results of Tukey test for D2, there was not any significant difference between Bego and Implantium. However, there was a significant difference in favor of Dio. Despite this, there were significant changes among the study groups for D3 (Table 3). 

**Table 3:** Tukey test results in comparing drills

	D2 (*p* Value)	D3 (*p* Value)
Dio & Bego	0.001	0.001
Dio & Implantium	0.006	0.001
Bego & Implantium	0.067	0.025

Histopathological evaluation with light microscope demonstrated a well-maintained bone structure consisting of plenty of osteocytes in the well-calcified matrix in Dio group (Figure 1). 

**Figure 2:**
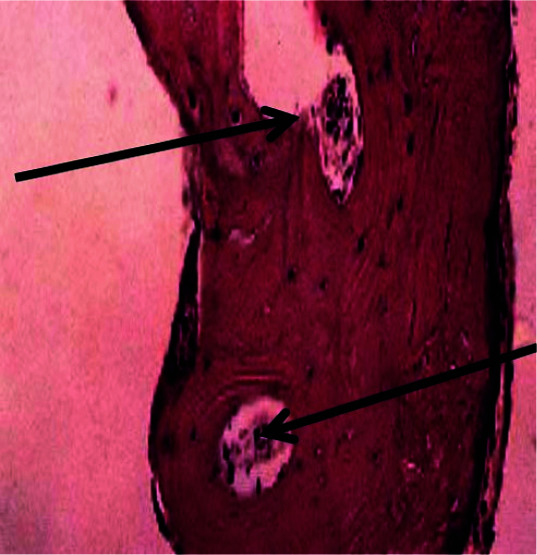
Histopathological view of Dio system drills

In samples obtained from Bego system drills, all samples were demonstrated as a complete secondary bone structure (Figure 2). 

**Figure 3:**
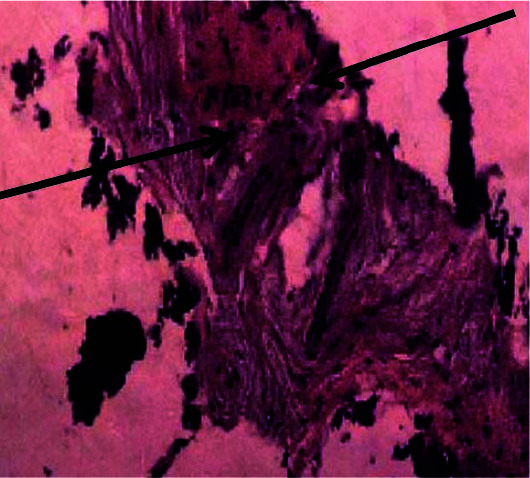
Histopathological view of Bego system drills

**Figure 4:**
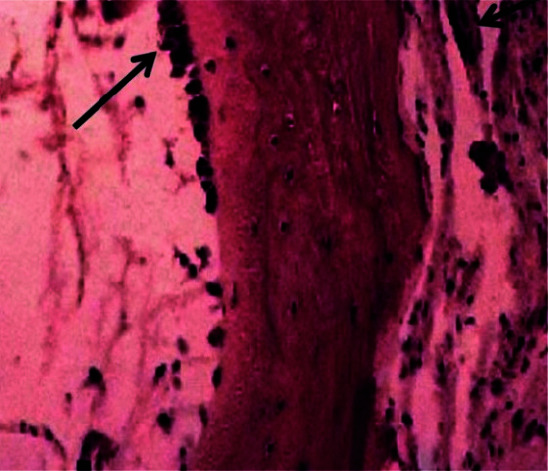
Histopathological view of Implantium system drills

The most significant bone changes were observed in Implantium samples. Normal osteocytes were seen with a diagnostic layer of covering osteoblasts in mature bones. Some cells were surrounded by a newly formed bony matrix indicating primary maturity stage of osteocytes. A basophilic line showed appositional growth and the presence of osteoprogenitor or osteogenic cells were visible (Figures 3-4).

## Discussion

Autogenous bone graft is the best graft material that has some advantages such as osteogenicity, osteoconductivity, and osteoinduction [ 8 , 16 ]. The use of bone acquired during osteotomy of dental implant would omit a secondary surgery procedure and reduces the discomfort at surgery site. These yielded particles are combination of cortical and spongy bone, the most important structure of which is osteocytes in calcified matrix [ 17 ].

In an animal study, researchers concluded that the collected bone presented more osteoactivity and absorption characteristics [ 18 ]. When these collected bony particles were grafted into dehiscence or fenestration implant sites, the results were successfully stable and well maintained [ 17 , 19 ].

According to our study, Dio drill samples showed more viability than the other groups. Histopathological study of Dio group demonstrated a well-maintained bony structure consisting of abundant osteocytes in well-calcified matrices. Histopathological results for Bego group showed more inflammation and the cell viability values were significantly less than Dio but the same as Implantium systems. Histopathological results for Implantium group showed more tissue changes and the cell viability values were significantly less than Dio but the same as Bego systems. Osteoblasts viability was significantly better in Dio group based on D2 and D3 evaluation.D3 bone has more porosity than D2, so it is less involved with heat and tissue necrosis. Based on all of these, more viability of D3 was predictable. Two main factors regarding grafting materials are their particle size and the available bone. In general, smaller particles are preferred in view of more absorption rate, extended contact area, and increased osteogenesis. Regardless of all these, there are some disadvantages such as absence of space for migration and proliferation of cells to vessels and bone [ 20 - 21 ].

Shapoff *et al*. [ 21 ] suggested 300-500μm as appropriate size. Urist *et al* suggested that the size range of 250-420 μm induces better ossification than 1000-2000μm. Whereas other study reported, bone particles of 125-1000 μm are the best size [ 22 ].

Park *et al*. [ 23 ] reported that low drilling speed leads to a large particle size. Marzook *et al*. [ 24 ] suggested that high speed drilling of dense bone in addition to irrigation results in less temperature change and better cell viability. Moreover, bone viability is better maintained while using lower speed. In brief, the drill diameter is inversely related to temperature changes [ 23 ].

In a systematic review, low-speed drilling without irrigation was compared with conventional drilling. It was concluded that low-speed drilling without irrigation presented better results [ 25 ]. 

There are three reasons for significant better success rates of smaller particles compared to large ones; they include increased surface area, increased osteoclasts activity and osteogenic activity, and finally osteogenesis stimulation [ 23 ]. The different drill geometry designs, such as web, thinning, and flute, may influence the bone formation. In this study, we used straight tapered Bego drills, parallel twist Implantium drills, and twiststepped-tapered Dio drills. These implant system drills have detailed characteristics presented in Table 3. 

The lesser web diameter results in better formation and discharge of bone chips; this is the same as the results of our study considering the better viability values and histopathological structure. Park *et al*. [ 23 ] reported that drilling geometry is one of the most important factors influencing bone chips size. Based on the results of our study, Dio drill was unfavorable for collecting bone chips. While there was not any relation between viability values, MTT evaluation showed better viability in Dio drill. Perhaps this is related to small bone particle sizes [ 23 ].

Bone debris particles are similar to tight spirals during osteotomy indicating metal drilling. Bone chips are created by separate fractures with local shape change in drill edges [ 26 ]. Increased flute number causes web drill strength. Increased web drill results in decreased flute width. Web diameter is a determining factor for flute volume and drill power, so narrow web is better for formation and gathering bone chips [ 26 ]. Large flute width is favorable for better bone chips collecting. Increased number of flutes is related to decreased flute width, so it is not suitable for formation and collecting bone chips. The web thinning decreases the cutting resistance and leads the bone chips and water from drill edge to flute. Thinning is also related to decreased drill fracture during drilling [ 26 ]. 

Different drills geometry can be effective on bone chips size during drilling. The drill design is allied to some factors such as cut efficiency, power, vibration, and bone chips formation. Deliberating the drill design is crucial to predict acquired bone particles volume for dehiscence and fenestration treatment [ 23 - 24 , 26 ].

Finally, Chen *et al*. [ 27 ] suggest that faster new bone formation of implant site is related to a reduced zone of osteocyte death that should be considered.

Despite the fact that piezosurgery is contributed with greater cell viability compared to traditional drills, some aspects such as speed of surgery may affect the technique selection for implant site osteotomy [ 28 ]. Chen *et al*. [ 29 ] introduced a novel osteotomy preparation technique to preserve implant site viability and enhance osteogenesis. Unique design of the cutting flutes with no irrigation and low-speed was used.

The results of the present study may also be used to design implant drills for better cell viability based on the used drills characteristics. Dio drills are twist, stepped, and tapered. We suggest assessing different drilling speeds by different implant systems for future studies. It is also better to use control drills in these studies.

## Conclusion

It may be concluded that drill geometry significantly influenced the viability of bone particles collected during the preparation of implant sites. Moreover, characteristic geometry alone cannot represent the performance of a particular drill, and several geometric features should be concerned. The results of this study showed that the geometry of the Dio drill was the best considering the viability and histopathological evaluations.

## Conflict of Interest

The authors declare that they have no conflict of interest.
